# Feasibility and Acceptability of Online Recruitment and an Online Brief Mindfulness Intervention Among Patients With Sickle Cell Disease

**DOI:** 10.7759/cureus.35073

**Published:** 2023-02-16

**Authors:** Dominic Arjuna B Ugarte, Adam Hanley, Jeffery A Dusek, Sarah Martin, William Cumberland, Sean Young

**Affiliations:** 1 Emergency Medicine, University of California Irvine, Orange County, USA; 2 Psychology, College of Social Work, The University of Utah, Salt Lake City, USA; 3 Integrative/Complementary Medicine, Connor Whole Health, University Hospitals Cleveland Medical Center, Cleveland, USA; 4 Anesthesiology, University of California Irvine, Orange County, USA; 5 Biostatistics, University of California, Los Angeles, Los Angeles, USA

**Keywords:** mindfulness-based therapy, sickle cell crisis, pain rating, sickle cell disease (scd), mindfulness meditation

## Abstract

Background

Pain among young adult patients with sickle cell disease (PWSCD) is a highly significant public health problem associated with reduced quality of life. Due to issues uniquely affecting PWSCD, including distrust of research, challenging life situations, debilitating pain, stigma, and logistical challenges (e.g., child or elder care and transportation), SCD researchers often find it challenging to meet sample size and enrollment targets. To our knowledge, all known SCD studies have solely recruited participants in person (e.g., clinics and local organizations) and utilized lengthy interventions with suboptimal recruitment and retention results. Newer recruitment methods, such as online recruitment, need to be explored for research among PWSCD. In this pilot project, we sought to address these challenges by investigating: 1) a novel method of using online outreach to recruit/enroll young PWSCD and 2) a novel, brief online mindfulness intervention adapted from Mindfulness-Oriented Recovery Enhancement (MORE): Mini-MORE designed to treat pain among PWSCD.

Methods

Participants (N = 32) were recruited online (e.g., paid advertisements on Facebook and NextDoor; free advertisements on Facebook groups and Reddit) and screened by phone from October 25 to November 8, 2022. Participants watched an online Mini-MORE video. Immediately before and after watching the video, participants rated their pain intensity and unpleasantness on an 11-point numeric rating scale (NRS). Afterward, participants were emailed an individualized link to additional audio recordings for ongoing practice. Immediately before and after accessing the additional recordings, participants rated their pain intensity, anxiety, and depression on an 11-point NRS. T-tests were used to examine the impact of Mini MORE on outcomes at two-time points.

Results

A total of 84 participants completed the self-screening. The majority of self-screeners resulted from free posts in Facebook groups (77%), Reddit (1%), and Craigslist (6%). Thirty-two (32) eligible participants agreed to join a Zoom meeting to watch the video. The entire Mini-MORE video was viewed by 31 of 32 participants (97%). Pain intensity decreased by 1.7 points (p<0.001, Cohen’s *d*=2.19), and pain unpleasantness decreased by 2.1 points (p<0.001, Cohen’s *d*=2.20). Listening to the supplemental recordings was associated with significant, immediate decreases in pain intensity by 1.3 points (p=0.015, Cohen’s *d*=2.05), anxiety by 1.8 points (p=0.022, Cohen’s *d*=3.10), and depression by 1.74 points (p=0.019, Cohen’s *d*=2.94).

Conclusions

Results suggest that online methods are feasible in recruiting and enrolling young PWSCD, and the online Mini-MORE intervention is acceptable among PWSCD. Future research is needed to assess whether Mini-MORE is associated with decreased pain symptomology in young PWSCD.

## Introduction

Pain is the most common complication of sickle cell disease (SCD), including severe acute pain episodes primarily from vaso-occlusive crises and chronic persistent pain [1-3]. Pain among young adult (18-30 years old) patients with SCD (PWSCD) is a significant public health problem associated with high rates of hospitalizations, ER visits, poor functional and psychosocial outcomes, and an increased rate of mortality [4-5]. By the time PWSCD reach adulthood, more than 55% have pain for more than half of the days, and almost a third report pain on 95% of days [6]. Despite their known side effects and potential for misuse, opioid medications remain the primary pharmacological treatment for SCD pain [6-8]. PWSCD need new treatment options that are safe and efficacious. Mindfulness-based interventions (MBIs) represent a promising and efficacious non-pharmacological pain treatment option [9-14] but have not been used to treat SCD pain. For example, Mindfulness-Oriented Recovery Enhancement (MORE) [15] has improved pain-related outcomes in three large randomized clinical trials (RCTs) involving chronic pain patients (N=250, N=115, N=95) [10,13-14]. In the latest study, participants receiving MORE demonstrated a significant 24% decrease in pain interference nine months after treatment ended (d=0.60). Similarly, 22% decreases in pain interference were observed in earlier MORE RCTs (e.g., d=0.78). However, the traditional eight-week MORE format requires too much time and resource investment for many patients and providers. To improve MORE's accessibility, the most therapeutic elements of the traditional eight-week MORE treatment have been distilled into a single, 20-minute intervention called Mini MORE. Mini MORE has effectively reduced surgical patients' pain in two large RCTs (N=285 and N=118) [16-17] and hospitalized patients' pain in a third RCT (N=244) [18]. Engaging PWSCD in a study of Mini MORE is challenging for multiple reasons. Due to issues uniquely affecting PWSCD, including distrust of research, challenging life situations, debilitating pain, stigma, and logistical challenges (e.g., child or elder care and transportation) [19-20], SCD researchers often find it challenging to meet sample size needs and enrollment timeline targets [21]. This recruitment challenge may result from limitations inherent to traditional recruitment methods. To our knowledge, all known SCD studies to date have recruited participants in person (e.g., through onsite referrals from clinics or organizations) [21]. Geographic constraints limit this approach (e.g., a finite number of PWSCD live in any given region), and the traditional, grounded/in-person approach risks pitting researchers against each other as they often recruit from the same potential pool of participants. Furthermore, the grounded recruitment approach excludes individuals who are not already connected with SCD clinics and organizations, likely those who are in the most need of additional support. The grounded approach might also be limited in reaching participants in rural/spread out areas who live far from clinics and organizations.

Online recruitment (e.g., free and paid advertisements on Facebook, Google, Craigslist, etc.) may offer one solution that can be used to improve enrollment in SCD studies. Researchers have utilized online recruitment for more than 15 years for various studies, especially among communities of color [22-23]. For example, Facebook, Instagram, Craigslist, and Reddit, have all been successfully and extensively used to recruit Black/African American and Latinx participants to studies [24-27]. These methods might be similarly applied to recruit and enroll PWSCD. To address these issues, we conducted a pilot study where we sought to assess the feasibility of using online methods to recruit PWSCD and investigate the acceptability of Mini MORE with PWSCD. A preliminary assessment of the effect of Mini MORE on pain intensity and pain-related symptomatology (i.e., pain unpleasantness, anxiety, depression) was also conducted.

## Materials and methods

Recruitment

Participants (N = 32) were recruited from October 25 to November 8, 2022, using online advertisements on platforms, including paid ads on Facebook and Nextdoor, as well as free ads on Facebook groups and Reddit. Ads were designed to target those in the United States living with SCD. When potential participants clicked on an ad, they were directed to a Qualtrics survey to check for eligibility. Eligibility criteria for participants were: 18-30 years old, US resident, and self-reported a diagnosis of SCD. Those who were not eligible were thanked for their interest and told their information would not be used and would be destroyed for their confidentiality. Those eligible were called to confirm they were a unique person (e.g., remove SPAM and bots attempting to enroll) and to set up a Zoom meeting to a further screen to confirm eligibility. If eligible and enrolled in the study, participants were then shown the Mini MORE video during the Zoom meeting. A total of 71 eligible PWSCD were initially screened over the phone for the study, and 32 participants agreed to and completed our Zoom meeting. The Zoom meeting was done with cameras on and was recorded for later analysis and transcription.

Mini MORE

Mini MORE is a 20-minute distilled adaptation of the traditional multi-week MORE program, which has been investigated in three previous RCTs. The Mini MORE video includes an introduction to pain neuroscience (2 minutes), an introduction to mindfulness (3 minutes), a mindfulness meditation (10 minutes), question prompts to facilitate mindfulness practice reflection (2 minutes), and strategies for integrating mindfulness practice into daily life (3 minutes). During the Zoom meeting, participants were provided with an individualized link to the Mini MORE video and survey (or had the study team stream the Mini MORE video for them over Zoom). Immediately before and after the video, participants were asked to rate their pain intensity and pain unpleasantness. After the meeting, participants were emailed an individualized link to audio recordings of supplemental practices (e.g., guided meditations on the breath, mindful reappraisal, and savoring) and were encouraged to use the recordings as much as they wanted. Pain intensity, anxiety, and depression were measured immediately before and after the supplemental practice recordings. Participants received a $10 Amazon gift code for completing the initial screening over the phone and a $20 Amazon gift code for completing the Zoom meeting and watching the Mini MORE video. No compensation was provided for listening to the supplemental practice recordings.

Measures

We included measures of pain intensity (“How much pain do you have?” [28]), pain unpleasantness (“How unpleasant is your pain?” [28]), anxiety (“How nervous, anxious, or on edge do you feel?” [29]), and depression (“How down, depressed, or uninterested in life do you feel? [29]) with individual items rated on a 0-10 numeric rating scale (NRS). The surveys for the initial video looked at pain intensity and unpleasantness, while the surveys for the supplemental recordings looked at pain intensity, anxiety, and depression. The NRS is a widely used and validated approach to measuring clinical pain and related symptomatology [16,18,30]. The anxiety and depression items were adapted from the GAD-2 and PHQ-2 [31-32]. To assess the feasibility and acceptability of Mini MORE, we looked at how many participants viewed the whole video and how many participants accessed the additional supplementary recordings. We expected that at least 80% would view the entire initial Mini MORE video and at least 50% would view the additional recordings.

Statistical analysis

To examine the impact of Mini MORE on outcomes at two time points, we conducted a series of paired samples t-tests. First, we conducted two paired sample t-tests using pain intensity and pain unpleasantness scores from immediately before to immediately after Mini MORE as the dependent variables. Second, we conducted three paired sample t-tests using pain intensity, anxiety, and depressive state scores from immediately before to immediately after the follow-up guided meditation as the dependent variables. For all analyses, p-values <0.05 were considered statistically significant, and Cohen’s d-values of 0.20, 0.50, and 0.80 were considered small, medium, and large effects, respectively. All statistical analyses were conducted in SPSS 27 (IBM Corp., Armonk, NY).

Ethics

This study was determined to be exempt by the University of California, Irvine Institutional Review Board. Some research at UCI is eligible for exempt self-determination. Please see the UCI Office of Research website: https://research.uci.edu/wp-content/uploads/confirmation-of-exempt-reference.pdf.

## Results

**Figure 1:**
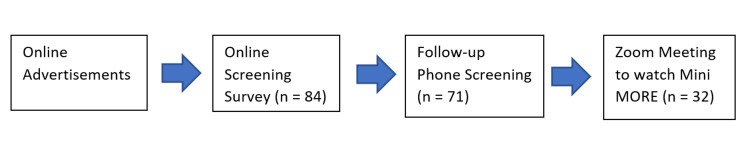
Participants flowchart.

A total of 84 participants completed the self-screening, with 10% and 6% coming from paid Facebook and Nextdoor ads, respectively. The majority came from free posts in Facebook groups (77%), Reddit (1%), and Craigslist (6%). A total of 71 (84% of self-screeners) completed the phone follow-up screening, with 32 participants (45% of those screened by phone) agreeing to join a Zoom meeting. Of the 32 participants participating in the Mini MORE Zoom meeting, the mean age was 26, the majority were male (N = 19), and all but one identified as Black or African American. Figure 1 and Table 1 show additional detail.

**Table 1:** Demographics of study population.

	Category	Interviewee
n		32
Age (mean, SD)		26.06 (2.96)
Race (%)	Black or African American	31 (96.9)
	Other: Hispanic	1 (3.1)
Ethnicity (%)	Non-Hispanic or Latino	23 (71.9)
	Hispanic or Latino	9 (28.1)
Gender Identity (%)	Female	13 (40.6)
	Male	19 (59.4)

The entire Mini MORE video was viewed by 31 of 32 participants (97%). Complete data were available for 29 participants. Table 2 presents mean scores for each dependent variable by assessment point for those viewing the whole video. In the week following their introduction to Mini MORE, 15 (51.7%) participants used their individualized link to access additional five-minute mindfulness practices. Of those 15 participants, 10 accessed a mindfulness practice once, and five accessed a mindfulness practice an average of 3.2 times. In all, a follow-up mindfulness video was viewed 19 times. Complete data were available for each of the 19 views.

**Table 2:** Pre- and post-assessment mean values for dependent variables. MORE: Mindfulness-Oriented Recovery Enhancement.

Variable	Pre-assessment	Post-assessment	Mean change	t	DF	P-value	Cohen’s d
Mini MORE							
Intensity	6.45 (2.61)	4.79 (2.64)	1.66	4.07	28	< 0.001	2.19
Unpleasantness	6.83 (2.19)	4.69 (2.16)	2.14	5.24	28	< 0.001	2.20
Follow-Up							
Intensity	8.05 (2.20)	6.79 (2.57)	1.26	2.69	18	0.015	2.05
Anxiety	8.37 (1.54)	6.58 (2.41)	1.79	2.515	18	0.022	3.10
Depression	8.16 (2.12)	6.42 (2.32)	1.74	2.57	18	0.019	2.94

T-test results revealed that Mini MORE was associated with significant decreases in pain intensity (p<0.001, Cohen’s d=2.19) and pain unpleasantness (p<0.001, Cohen’s d=2.20) from immediately before to immediately after the intervention. Pain intensity decreased by 1.7 points, and pain unpleasantness decreased by 2.1 points. T-test results revealed that listening to the follow-up guided meditations was associated with significant decreases in pain intensity (p=0.015, Cohen’s d=2.05), anxiety (p=0.022, Cohen’s d=3.10), and depressive state (p=0.019, Cohen’s d=2.94). Pain intensity decreased by 1.3 points, anxiety decreased by 1.8 points, and depression decreased by 1.7 points.

## Discussion

This pilot study investigated a novel method of recruiting young PWSCD and a novel method for providing the intervention online. Results suggest that online recruitment is an effective method of recruiting PWSCD, with 71 PWSCD enrolling in about three weeks. As such, online recruitment and intervention delivery may prove an essential tool for reaching and treating PWSCD. Not only can online methods reduce some of the barriers inherent to traditional, in-person recruitment and treatment, but they can also reach a wider population of PWSCD, critically those in areas without ready access to care. Online methods may also reduce research and treatment costs. While we did use paid ads on platforms like Nextdoor, approximately 84% of the 71 enrolled participants came from free advertisements on Facebook groups, Reddit, and Craigslist, demonstrating the cost-effectiveness of online outreach among PWSCD. Our results also further suggest that young PWSCD find the online Mini MORE intervention acceptable, and Mini MORE involvement is associated with decreased pain and pain-related clinical symptomatology in young PWSCD. With respect to acceptability, 97% of participants viewed the full video, and 15 participants accessed their individualized link to supplemental practices during the week after watching the video without further support or prompting. With respect to pain, there was a significant decrease in both pain intensity and pain unpleasantness after participating in the first Mini MORE video. Results indicated similar significant effects on pain intensity, anxiety, and depression following the use of the additional mindfulness recordings. Collectively, the decreases associated with Mini MORE involvement in the current study, including PWSCD, are comparable to, or greater than, those observed in our previous RCTs of Mini MORE [16-18]. For example, in total joint arthroplasty patients, preoperative pain intensity from joint disease was immediately reduced by 24%, and pain unpleasantness was immediately reduced by 29% [16]. In this pilot study, pain intensity was immediately reduced by 26%, and pain unpleasantness was immediately reduced by 31%. However, as there was no control group, it is possible that attention from the research team or distraction may have accounted for the observed effects. Future RCTs are now needed to investigate Mini MORE’s clinical utility (i.e., if the effect is long-lasting). 

Despite notable strengths, study limitations should also be noted. First, a lack of input from PWSCD in the initial development of Mini MORE may hamper its acceptability and clinical impact. Although Mini MORE has been acceptable to other clinical populations [16-18] and was found to be acceptable in this pilot study, interviewing PWSCD exposed to Mini MORE will provide important insights into how to adapt Mini MORE for PWSCD better. Second, since recruitment targeted younger people (18-30 years old) through social media, participants in this study may only represent some PWSCD. To ensure Mini MORE’s generalizability, it would be useful to introduce Mini MORE to PWSCD not recruited online and deliver it in a grounded format. Third, while we did have a two-step screening process for our participants and asked them about their SCD pain, we did not have any lab tests to confirm SCD diagnosis. Self-report procedures, however, are standard in online studies and, if anything, may underestimate the public health issue (e.g., HIV serostatus self-reporting is standard) [33-35]. Requiring to show the diagnosis may also create a bias toward only those who are able to and comfortable sharing their health documents. Furthermore, sharing health documents also increases privacy risks for the participants and is not covered under our self-exempt protocol. Finally, the small sample size threatens internal and external validity; replication in a larger sample is needed. A larger sample size will also be needed to test the effectiveness of Mini MORE. Traditional mindfulness training methods commonly encourage new practitioners to seek out a community that will support their practice continuity through guidance and accountability [36-38]. The next steps will be to examine whether Mini MORE is capable of encouraging sustained improvements in SCD pain. Sustained improvement following Mini MORE involvement is likely to result from sustained mindfulness practice. Future studies may wish to encourage sustained mindfulness practice with a community support group model, such as those used in the Harnessing Online Peer Education (HOPE) intervention [22,24-25]. 

## Conclusions

Results suggest that online methods are feasible in recruiting and enrolling young adult PWSCD. They found the online Mini MORE intervention acceptable based on a 97% watch rate of the full video and 51.7% utilizing additional recordings. Future research is needed to assess whether Mini MORE is associated with decreased pain symptomatology in young PWSCD.
